# Glycemic Control Trajectories and Risk of Perinatal Complications Among Individuals With Gestational Diabetes

**DOI:** 10.1001/jamanetworkopen.2022.33955

**Published:** 2022-09-29

**Authors:** Rana F. Chehab, Assiamira Ferrara, Mara B. Greenberg, Amanda L. Ngo, Juanran Feng, Yeyi Zhu

**Affiliations:** 1 Division of Research, Kaiser Permanente Northern California, Oakland; 2 Department of Obstetrics and Gynecology, Kaiser Permanente Northern California, Oakland; 3 Regional Perinatal Service Center, Kaiser Permanente Northern California, Santa Clara; 4 Department of Epidemiology and Biostatistics, University of California, San Francisco

## Abstract

**Importance:**

Glycemic control is the cornerstone of gestational diabetes management. Glycemic control trajectories account for differences in longitudinal patterns throughout pregnancy; however, studies on glycemic control trajectories are scarce.

**Objective:**

To examine whether glycemic control trajectories from gestational diabetes diagnosis to delivery were associated with differential risk of perinatal complications.

**Design, Setting, and Participants:**

This population-based cohort study included individuals with gestational diabetes with longitudinal electronic health record data from preconception to delivery who received prenatal care at Kaiser Permanente Northern California (KPNC) and were enrolled in KPNC’s telemedicine-based gestational diabetes care program between January 2007 and December 2017. Data analysis was conducted from September 2021 to January 2022.

**Exposures:**

Glycemic control trajectories were derived using latent class modeling based on the American Diabetes Association’s recommended self-monitoring of blood glucose measurements. Optimal glycemic control was defined as at least 80% of all measurements meeting the targets at KPNC clinical settings.

**Main Outcomes and Measures:**

Multivariable Poisson regression models were used to estimate the associations of glycemic control trajectories with cesarean delivery, preterm birth, shoulder dystocia, large- and small-for-gestational-age, and neonatal intensive care unit admission and stay of 7 days or longer.

**Results:**

Among a total of 26 774 individuals (mean [SD] age, 32.9 [5.0] years; 11 196 Asian or Pacific Islander individuals [41.8%], 1083 Black individuals [4.0%], 7500 Hispanic individuals [28.0%], and 6049 White individuals [22.6%]), 4 glycemic control trajectories were identified: stably optimal (10 528 individuals [39.3%]), rapidly improving to optimal (9151 individuals [34.2%]), slowly improving to near-optimal (4161 individuals [15.5%]), and slowly improving to suboptimal (2934 individuals [11.0%]). In multivariable models with the rapidly improving to optimal trajectory group as the reference group, glycemic control trajectories were associated with perinatal complications with a gradient across stably optimal to slowly improving to suboptimal. For individuals in the stably optimal trajectory group, there were lower risks of cesarean delivery (adjusted relative risk [aRR], 0.93 [95% CI, 0.89-0.96]), shoulder dystocia (aRR, 0.75 [95% CI, 0.61-0.92]), large-for-gestational age (aRR, 0.74 [95% CI, 0.69-0.80]), and neonatal intensive care unit admission (aRR, 0.90 [95% CI, 0.83-0.97]), while for patients in the slowly improving to suboptimal glycemic control trajectory group, risks were higher for cesarean delivery (aRR, 1.18 [95% CI, 1.12-1.24]; (*P* for trend < .001), shoulder dystocia (aRR, 1.41 [95% CI, 1.12-1.78]; *P* for trend < .001), large-for-gestational-age (aRR, 1.42 [95% CI, 1.31-1.53]; *P* for trend < .001), and neonatal intensive care unit admission (aRR, 1.33 [95% CI, 1.20-1.47]; *P* for trend < .001). The risk of small-for-gestational-age was higher in patients in the stably optimal group (aRR, 1.10 [95% CI, 1.02-1.20]) and lower in the slowly improving to suboptimal group (aRR, 0.63 [95% CI, 0.53-0.75]).

**Conclusions and Relevance:**

These findings suggest that slowly improving to near-optimal and slowly improving to suboptimal glycemic control trajectories were associated with increased risk of perinatal complications. Future interventions should help individuals achieve glycemic control early after gestational diabetes diagnosis and throughout pregnancy to decrease the risk of perinatal complications.

## Key Points

**Question:**

Among individuals with gestational diabetes, are distinct glycemic control trajectories from gestational diabetes diagnosis to delivery associated with differential risk of perinatal complications?

**Findings:**

In this cohort study of 26 774 individuals with gestational diabetes, 4 glycemic control trajectories (stably optimal, rapidly improving to optimal, slowly improving to near-optimal, and slowly improving to suboptimal) were identified. A gradient of increasing risk of cesarean delivery, shoulder dystocia, large-for-gestational-age, and neonatal intensive care unit admission was observed across stably optimal to slowly improving to suboptimal glycemic control trajectories.

**Meaning:**

These findings suggest that gestational diabetes interventions should help individuals achieve glycemic control early after diagnosis and throughout pregnancy to decrease the risk of perinatal complications.

## Teaser

This cohort study examines glycemic control trajectories in gestational diabetes and their associations with risk of perinatal complications.

## Introduction

Gestational diabetes, one of the most common pregnancy complications,^1^ affects around 8% of pregnancies each year in the US.^2^ Gestational diabetes is associated with a higher risk of adverse perinatal outcomes, including hypertensive disorders of pregnancy, cesarean delivery, macrosomia, shoulder dystocia, neonatal hypoglycemia, and increased long-term risk of diabetes and cardiovascular disease.^3^ Randomized clinical trials of treatment of gestational diabetes using medical nutrition therapy and insulin therapy reduced the rate of perinatal complications,^4,5^ suggesting that glycemic control may improve perinatal outcomes.

The essential role of glycemic control in gestational diabetes management has been widely acknowledged, with the American Diabetes Association (ADA) recommending the use of self-monitoring of blood glucose as the primary measure of glycemic control during pregnancy.^6^ However, large-scale, population-based data on glycemic control profiles based on serial self-monitoring of blood glucose measurements and their associations with perinatal complications among individuals with gestational diabetes are sparse.^7^ Furthermore, conventional dichotomization of glycemic control status (ie, optimal vs suboptimal) does not capture the progressive changes of glycemic control during pregnancy. Thus, studies of glycemic control trajectories, which account for differences in glycemic control patterns throughout pregnancy and profile the time-specific glycemic control status and its longitudinal trends following gestational diabetes diagnosis to delivery, are warranted.

To better inform gestational diabetes management, we conducted a population-based study of 26 774 individuals with gestational diabetes in an integrated health care system providing universal supplemental gestational diabetes care via a telemedicine program. We profiled distinct glycemic control trajectories after gestational diabetes diagnosis through delivery and examined whether the trajectories were differentially associated with the risk of perinatal complications. We also assessed whether individual-level multidomain factors were associated with the glycemic control trajectories to inform preventive and intervention strategies.

## Methods

This cohort study was approved by the KPNC institutional review board, which waived the requirement for informed consent from study individuals given the use of only electronic health record (EHR) data and the large sample size, which made it not possible to obtain authorization from each individual included in the study. The study followed the Strengthening the Reporting of Observational Studies in Epidemiology (STROBE) reporting guideline.

### Study Design

This is a longitudinal cohort study of individuals with gestational diabetes who received prenatal care between January 1, 2007, and December 31, 2017, at Kaiser Permanente Northern California (KPNC), an integrated health care delivery system serving 4.5 million members who are highly representative of the underlying population.^8,9^ Pregnant individuals at KPNC are universally screened (96%) for gestational diabetes at 24 to 28 weeks of gestation.^10^ Gestational diabetes is diagnosed via a 2-step approach (a 50-g, 1-hour glucose challenge test [GCT] followed by a diagnostic 100-g, 3-hour oral glucose tolerance test [OGTT]) according to the Carpenter and Coustan criteria, as recommended by the American College of Obstetricians and Gynecologists^11^ or according to the recommendations by the International Association of Diabetes and Pregnancy Study Groups and ADA.^6,12^ Individuals with diabetes prior to pregnancy were identified through the KPNC diabetes registry and excluded.^13^ Since 2007, individuals with gestational diabetes have been universally offered enrollment to a nurse-based management program providing supplemental care via telemedicine by the KPNC Regional Perinatal Service Center (RPSC).^6^ This program offers telephone counseling 7 days a week on diet, physical activity therapy, and glucose monitoring using a glucometer.

### Glycemic Control Assessment

We used self-monitoring of blood glucose measurements to assess glycemic control as recommended by the ADA.^6^ Individuals enrolled in the RPSC program are instructed to record self-monitored of blood glucose measurements 4 times per day: fasting before breakfast and 1 hour after breakfast, lunch, and dinner. The measurements are then reported to the nurses or dietitians during weekly counseling calls and recorded in the Patient Reported Capillary Glucose Clinical Database. Optimal glycemic control was defined based on RPSC clinical practice as at least 80% of all self-monitoring of blood glucose measurements meeting the ADA targets: less than 95 mg/dL for fasting and less than 140 mg/dL for 1-hour postprandial glucose (to convert to millimoles per liter, multiply by 0.0555).^14^ If optimal glycemic control is not achieved over a 2-week period, the gestational diabetes treatment plan is intensified.

### Perinatal Complications

Data on the following perinatal complications were collected from the EHR: cesarean delivery, preterm birth (<37 weeks of gestation), shoulder dystocia, admission to neonatal intensive care (NICU), NICU stay of 7 days or longer, stillbirth, and sex- and gestational age–specific birthweight categories based on a 2017 US reference population (small-for-gestational age [SGA], <10th percentile; appropriate-for-GA, 10th-90th percentile; and large-for-GA [LGA], >90th percentile]).^15^

### Individual-Level Multidomain Factors

Data on individual-level factors across 3 domains were obtained from the EHR: sociodemographic, clinical, and lifestyle factors; indicators of gestational diabetes severity; and indicators of gestational diabetes management adherence. Sociodemographic factors included age at delivery, race and ethnicity, Medicaid or Medicare insurance during pregnancy, and neighborhood poverty level (quartiles) derived using an individual’s probability of falling below the poverty threshold levels based on the percentage below the poverty level in their neighborhood census block group using individual’s address as recorded in the EHR.^16^ Race and ethnicity were self-reported and categorized as Hispanic, non-Hispanic Asian or Pacific Islander, non-Hispanic Black, non-Hispanic White, and other. The other race and ethnicity category included American Indian or Alaskan Native individuals and those who identified as multiracial or with missing race and ethnicity, which were combined owing to small sample size. Race and ethnicity were included because of known associations with perinatal outcomes. Clinical and lifestyle factors included multiparity, prepregnancy body mass index (BMI; calculated as prepregnancy weight [measured closest to the last menstrual period within 12 weeks prior] in kilograms divided by height in meters squared [measured within 12 months before pregnancy]), smoking and alcohol use during pregnancy, and gestational weight gain (calculated using weight at last menstrual period and weight at delivery) compared with Institute of Medicine recommendations.^17^

Indicators of gestational diabetes severity included higher OGTT glucose levels (ie, ≥2 of the fasting, 1-hour, 2-hour, and 3-hour glucose levels during OGTT >1 SD above the respective population mean), early gestational diabetes diagnosis (ie, before 24 weeks of gestation), and gestational diabetes treatment modality. Indicators of gestational diabetes management adherence included engagement in the RPSC program (ie, completing ≥1 call biweekly with RPSC nurses between the RPSC program enrollment and delivery) and daily frequency of self-monitoring of blood glucose measurements.

### Statistical Analysis

Glycemic control trajectories were derived using latent class models^18^ fit using SAS Proc Traj version 2020^19,20^ (SAS Institute). Details on trajectory modeling are presented in the eAppendix and eTable 1 in the Supplement. To examine the associations between glycemic control trajectories, perinatal complications, and individual-level multidomain factors, we constructed multivariable Poisson regression models to estimate adjusted relative risks (aRRs) and 95% CIs. Generalized estimating equations were used to calculate the robust SEs that account for clustering due to multiple index pregnancies of the same participant during the study period. We constructed sequential multivariable models adjusting for sociodemographic, clinical, and lifestyle factors (model 1); additionally, indictors of gestational diabetes severity (model 2); and additionally, gestational diabetes management adherence indicators (model 3). For the first part of the analysis, the glycemic control trajectories served as the exposure and the perinatal complications were the outcome of interest. For the second part, the individual-level multidomain factors were the exposure and the glycemic control trajectories served as the outcome of interest.

To reduce bias due to missing data (ranging between 0.2% for neighborhood poverty level and 9.6% for alcohol during pregnancy), we used multiple imputation based on all covariates, exposures, and outcomes of interest to create 10 complete data sets and combined the analyses results on each complete data set using Rubin rule.^21^ *P* values were corrected according to the Benjamini-Hochberg procedure controlling the false discovery rate. *P* for trend was calculated using the Poisson trend test.^22^ A 2-sided *P* < .05 was considered significant. All analyses were performed with SAS version 9.4 (SAS Institute). Data analysis was conducted from September 2021 to January 2022.

## Results

### Characteristics of the Study Population

Among 30 597 individuals with gestational diabetes who received prenatal care at KPNC in 2007 to 2017, 26 774 individuals (mean [SD] age, 32.9 [5.0] years; 11 196 Asian or Pacific Islander individuals [41.8%], 1083 Black individuals [4.0%], 7500 Hispanic individuals [28.0%], and 6049 White individuals [22.6%]) were enrolled in the RPSC program and included in our analysis. A total of 9835 infants (35.1%) born to individuals with gestational diabetes were delivered via cesarean section, 2553 infants (9.5%) were born preterm, 601 infants (2.2%) had shoulder dystocia, 4050 infants (15.1%) were born LGA, 2588 infants (9.7%) were born SGA, 3127 infants (11.7%) were admitted to NICU, and 1120 infants (4.2%) had a NICU stay of at least 7 days (Table 1). Stillbirth after gestational diabetes diagnosis was rare (<0.1%) and not examined as a perinatal complication.

**Table 1.** Individual-Level Multidomain Factors and Perinatal Complications by Glycemic Control Trajectories Among Individuals With Gestational Diabetes Who Received Prenatal Care in 2007-2017

Factor	No. (%)				
	T1 (n = 10 528)	T2 (n = 9151)	T3 (n = 4161)	T4 (n = 2934)	Total (N = 26 774)
Age at delivery, y					
18-24	585 (5.6)	409 (4.5)	179 (4.3)	140 (4.8)	1313 (4.9)
25-29	2238 (21.3)	1764 (19.3)	743 (17.9)	574 (19.6)	5319 (19.9)
30-34	3959 (37.6)	3440 (37.6)	1543 (37.1)	1060 (36.1)	10 002 (37.4)
35-55	3746 (35.6)	3538 (38.7)	1696 (40.8)	1160 (39.5)	10 140 (37.9)
Race and ethnicity					
Asian or Pacific Islander	4667 (44.3)	3755 (41.0)	1710 (41.1)	1064 (36.3)	11 196 (41.8)
Black	273 (2.6)	382 (4.2)	222 (5.3)	206 (7.0)	1083 (4.0)
Hispanic	2805 (26.6)	2607 (28.5)	1181 (28.4)	907 (30.9)	7500 (28.0)
White	2455 (23.3)	2067 (22.6)	895 (21.5)	632 (21.5)	6049 (22.6)
Other^a^	328 (3.1)	340 (3.7)	153 (3.7)	125 (4.3)	946 (3.5)
Neighborhood poverty level quartile (% below threshold)^b^					
1 (0-4, lowest)	4084 (38.8)	3430 (37.5)	1570 (37.7)	977 (33.3)	10 061 (37.6)
2 (5-9)	2627 (25.0)	2255 (24.6)	1027 (24.7)	764 (26.0)	6673 (24.9)
3 (10-19)	2535 (24.1)	2262 (24.7)	1034 (24.8)	798 (27.2)	6629 (24.8)
4 (≥20, highest)	1257 (11.9)	1186 (13.0)	523 (12.6)	390 (13.3)	3356 (12.5)
Medicaid or Medicare during pregnancy	845 (8.0)	923 (10.1)	413 (9.9)	388 (13.2)	2569 (9.6)
Multiparity	5898 (56.0)	5327 (58.2)	2573 (61.8)	1940 (66.1)	15 738 (58.8)
Prepregnancy BMI					
<18.5	214 (2.0)	101 (1.1)	28 (0.7)	9 (0.3)	352 (1.3)
18.5-24.9	3221 (30.6)	1786 (19.5)	720 (17.3)	360 (12.3)	6087 (22.7)
25.0-29.9	3677 (34.9)	2972 (32.5)	1317 (31.7)	844 (28.8)	8810 (32.9)
≥30.0	3416 (32.4)	4292 (46.9)	2096 (50.4)	1721 (58.7)	11 525 (43.0)
Gestational weight gain^c^					
Below recommendations	4101 (42.8)	3092 (36.8)	1255 (32.5)	680 (25.1)	9128 (37.2)
As recommended	3099 (32.4)	2535 (30.2)	1249 (32.4)	776 (28.6)	7659 (31.2)
Above recommendations	2372 (24.8)	2775 (33.0)	1355 (35.1)	1259 (46.4)	7761 (31.6)
Smoking during pregnancy	183 (1.7)	248 (2.7)	101 (2.4)	140 (4.8)	672 (2.5)
Alcohol during pregnancy	608 (5.8)	589 (6.4)	303 (7.3)	232 (7.9)	1732 (6.5)
Higher OGTT glucose levels^d^	733 (7.0)	1234 (13.5)	575 (13.8)	579 (19.7)	3121 (11.7)
Early GDM diagnosis^e^	1962 (18.6)	1794 (19.6)	1545 (37.1)	1001 (34.1)	6302 (23.5)
RPSC program engagement^f^	1110 (10.5)	1039 (11.4)	495 (11.9)	364 (12.4)	3008 (11.2)
Frequency of SMBG measurements tertile (times/d)					
1 (≤2)	2805 (26.6)	2602 (28.4)	1420 (34.1)	1304 (44.4)	8131 (30.4)
2 (3)	3914 (37.2)	3430 (37.5)	1598 (38.4)	1031 (35.1)	9973 (37.2)
3 (≥4)	3809 (36.2)	3119 (34.1)	1143 (27.5)	599 (20.4)	8670 (32.4)
GDM treatment modality					
Lifestyle	9408 (89.4)	4584 (50.1)	1563 (37.6)	493 (16.8)	16 048 (59.9)
Oral medications	1081 (10.3)	4193 (45.8)	2209 (53.1)	1713 (58.4)	9196 (34.4)
Insulin therapy	39 (0.4)	374 (4.1)	389 (9.4)	728 (24.8)	1530 (5.7)
Cesarean delivery	3267 (31.0)	3273 (35.8)	1540 (37.0)	1305 (44.5)	9385 (35.1)
Preterm birth	919 (8.7)	860 (9.4)	382 (9.2)	392 (13.4)	2553 (9.5)
Shoulder dystocia	170 (1.6)	209 (2.3)	114 (2.7)	108 (3.7)	601 (2.2)
Birth weight categories^g^					
AGA	8177 (77.7)	6792 (74.2)	2992 (71.9)	1957 (66.7)	19918 (74.4)
LGA	1039 (9.9)	1417 (15.5)	775 (18.6)	819 (27.9)	4050 (15.1)
SGA	1230 (11.7)	868 (9.5)	352 (8.5)	138 (4.7)	2588 (9.7)
NICU admission	1025 (9.7)	1057 (11.6)	535 (12.9)	510 (17.4)	3127 (11.7)
NICU stay ≥7 d	412 (3.9)	376 (4.1)	163 (3.9)	169 (5.8)	1120 (4.2)

Abbreviations: AGA, appropriate-for-gestational age; BMI, body mass index (calculated as weight in kilograms divided by height in meters squared); GDM, gestational diabetes; LGA, large-for-gestational age; NICU, neonatal intensive care unit; OGTT, oral glucose tolerance test; RPSC, Regional Perinatal Service Center; SMBG, self-monitoring of blood glucose; SGA, small-for-gestational age; T1, stably optimal trajectory; T2, rapidly improving to optimal trajectory; T3, slowly improving to near-optimal trajectory; and T4, slowly improving to suboptimal trajectory.

^a^ Other race and ethnicity includes American Indian or Alaskan Native, multiracial, and missing race and ethnicity.

^b^ Defined as percentage of households in neighborhood below the poverty level, divided into quartiles.

^c^ Gestational weight gain between last menstrual period and delivery compared with Institute of Medicine recommendations.^17^

^d^ Higher OGTT glucose levels were defined as at least 2 of the fasting, 1-hour, 2-hour, and 3-hour glucose levels during the 100-g, 3-hour oral glucose tolerance test more than 1 SD above the respective population mean.

^e^ Early GDM diagnosis was defined as diagnosis of GDM before 24 weeks of gestation.

^f^ RPSC program engagement was defined as at least 1 call biweekly between enrollment into the RPSC management of glycemic control program and delivery.

^g^ Birthweight categories were derived using sex- and gestational age–specific percentiles calculated using a 2017 US reference population.

**Figure 1.**
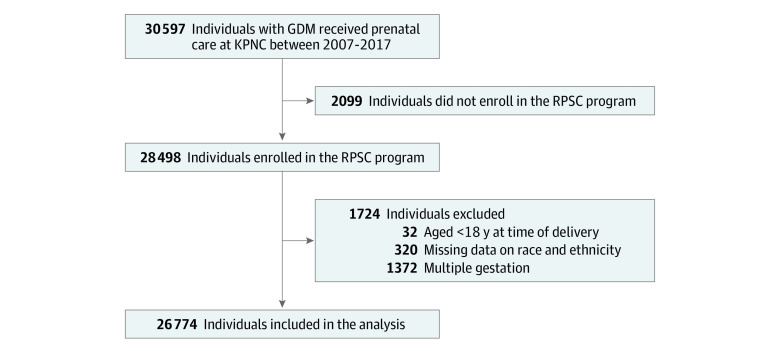
Study Participant Flowchart GDM, gestational diabetes; KPNC, Kaiser Permanente Northern California; RPSC, Regional Perinatal Service Center.

A total of 3823 individuals (12.5%) were excluded for the following reasons: 2099 did not enroll in the RPSC program, 32 were aged younger than 18 years at the time of delivery, 320 had missing data on race and ethnicity, and 1372 had nonsingleton gestations (Figure 1). Compared with the analytical sample of 26 774 individuals, individuals with gestational diabetes who did not enroll in the RPSC program were more likely to be younger, to be Black or Hispanic, to be multiparous, to have obesity before pregnancy, and to smoke during pregnancy and less likely to consume alcohol during pregnancy (eTable 2 in the Supplement).

### Glycemic Control Trajectories

**Figure 2.**
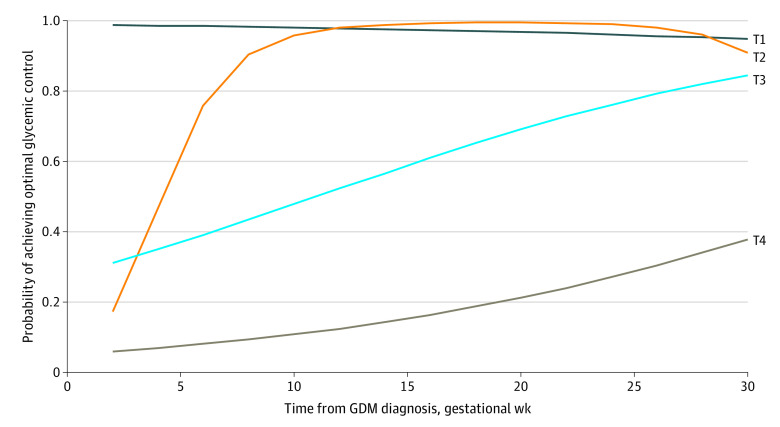
Glycemic Control Trajectories Between Gestational Diabetes Diagnosis and Delivery Trajectories T1-T4 were derived using serial self-monitored blood glucose measurements between gestational diabetes diagnosis and delivery. Optimal glycemic control was defined as at least 80% of all self-monitored blood glucose measurements meeting the targets recommended by the American Diabetes Association guidelines and implemented at Kaiser Permanente Northern California. T1 indicates stably optimal (10 528 individuals [39.3%]); T2, rapidly improving to optimal (9151 individuals [34.2%]); T3, slowly improving to near-optimal (4161 individuals [15.5%]); and T4, slowly improving to suboptimal (2934 individuals [11.0%]).

Individuals with gestational diabetes had a mean (SD) of 239.6 (160.6) self-monitored blood glucose measurements over 11.8 (6.6) weeks (eTable 3 in the Supplement). We identified 4 distinct glycemic control trajectories (Figure 2; eFigure in the Supplement). Trajectory 1 (T1) represented the stably optimal glycemic control trajectory with the largest proportion of individuals in our study (10 528 individuals [39.3%]), which started with a 1.0 probability of optimal glycemic control at gestational diabetes diagnosis and maintained that until delivery. T2 represented the rapidly improving to optimal glycemic control trajectory (9151 individuals [34.9%]), which started with less than 0.2 probability of achieving optimal glycemic control, reached optimal control by 8 weeks after diagnosis and maintained it except for a slight decrease during the last 2 weeks before delivery. T3 represented the slowly improving to near-optimal glycemic control trajectory (4161 individuals [15.5%]), which started with 0.3 probability of achieving optimal glycemic control and improved to more than 0.8 probability of achieving optimal glycemic control. Finally, T4 represented the slowly improving to suboptimal glycemic control trajectory (2934 individuals [11.0%]), which had a slowly improving glycemic control trajectory similar to T3 but started at a lower probability (<0.1) of achieving optimal glycemic control and improved to a probability of approximately 0.4. In sum, more than 90% of study individuals had improvements in their glycemic control across pregnancy, with almost 75% of them (in T1 and T2) reaching optimal control by 8 weeks after diagnosis.

### Glycemic Control Trajectories Associated With Perinatal Complications

**Figure 3.**
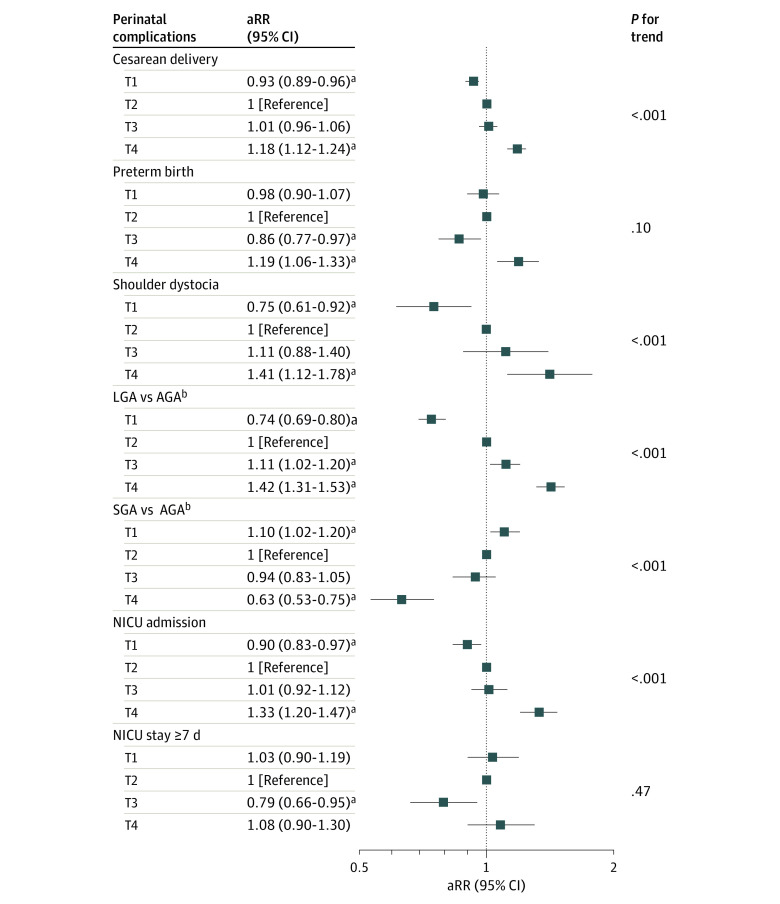
Associations of Glycemic Control Trajectories With Perinatal Complications Data are presented as adjusted relative risk (95% confidence interval) calculated using Poisson regression models with robust SEs. *P*-for-trend was calculated using the Poisson trend test. Model adjusted for age at delivery, race and ethnicity, neighborhood poverty level, Medicare or Medicaid during pregnancy, multiparity, prepregnancy body mass index, smoking and alcohol during pregnancy, higher oral glucose tolerance test levels, early gestational diabetes diagnosis, RPSC program engagement, and frequency of self-monitoring of blood glucose measurements. aRR indicates adjusted relative risk; AGA, appropriate-for-gestational age; LGA, large-for-gestational age; NICU, neonatal intensive care unit; SGA, small-for-gestational age; T1, stably optimal (10 528 individuals [39.3%]); T2, rapidly improving to optimal (9151 individuals [34.2%]); T3, slowly improving to near-optimal (4161 individuals [15.5%]); and T4, slowly improving to suboptimal (2934 individuals [11.0%]). ^a^False discovery rate–corrected *P* < .05. ^b^AGA, LGA, and SGA birthweight categories were derived using sex and gestational age-specific percentiles calculated using a 2017 US reference population.

Figure 3 shows the aRRs of the associations between glycemic control trajectories and perinatal complications. We used T2 as the reference trajectory since it represented a subgroup of individuals with gestational diabetes showing the greatest improvement in glycemic control and thus reflected the largest benefit associated with the RPSC program. Overall, the point estimates of the associations with perinatal complications remained similar, with slight attenuation after sequential adjustment for sociodemographic, clinical, and lifestyle factors (model 1); indicators of gestational diabetes severity (model 2); and gestational diabetes management adherence (model 3) (eTable 4 in the Supplement).

In the fully adjusted model 3, we observed a gradient of associations across glycemic control trajectories (from T1 to T4) with the following perinatal complications: cesarean delivery, shoulder dystocia, LGA, SGA, and NICU admission (Figure 3). Specifically, the risk of cesarean delivery increased across T1 to T4, with T1 showing a lower risk (aRR, 0.93 [95% CI, 0.89-0.96]) and T4 showing a higher risk (aRR, 1.18 [95% CI, 1.12-1.24]) compared with T2 (*P* for trend < .001). Similarly, the risk of shoulder dystocia and NICU admission increased across T1 to T4, with T1 showing a lower risk (shoulder dystocia: aRR, 0.75 [95% CI, 0.61-0.92]; NICU admission: aRR, 0.90 [95% CI, 0.83-0.97]) and T4 showing a higher risk (shoulder dystocia: aRR, 1.41 [95% CI, 1.12-1.78]; *P* for trend <.001; NICU admission: aRR, 1.33 [95% CI, 1.20-1.47]; *P* for trend <.001). The risk of LGA increased across trajectories (T1: aRR, 0.74 [95% CI, 0.69-0.80]; T3: aRR, 1.11 [95% CI, 1.02-1.20]; T4: aRR, 1.42 [95% CI, 1.31-1.53]; *P* for trend < .001) compared with T2, whereas the risk of SGA decreased, with T1 showing a higher risk (aRR, 1.10 [95% CI, 1.02-1.20]) and T4 showing a lower risk (aRR, 0.63 [95% CI, 0.53-0.75]; *P *for trend < .001).

The risk of preterm birth and a NICU stay of 7 days or longer did not show a significant trend across T1 to T4. Compared with T2, the risk of preterm birth was lower in T3 (aRR, 0.86 [95% CI, 0.77-0.97]) and higher in T4 (aRR, 1.19 [95% CI, 1.06-1.33]; *P* for trend = .10) and the risk of a NICU stay of 7 days or longer was lower in T3 (aRR, 0.79 [95% CI, 0.66-0.95]; *P* for trend = .47).

We conducted several sensitivity analyses to ensure the robustness of our findings. First, we examined the associations by preterm birth subtype. The positive association of T4 vs T2 with medically indicated preterm birth was more pronounced compared with spontaneous preterm birth (eTable 4 in the Supplement). Second, we examined the associations using T1 as the reference trajectory; similar patterns were observed to those using T2 as the reference trajectory (eTable 5 in the Supplement). Third, we adjusted for gestational weight gain in the multivariate models, although it is potentially on the causal pathway between glycemic control and perinatal complications (eTable 6 in the Supplement). Fourth, we excluded individuals diagnosed with gestational diabetes before 24 weeks of gestation (eTable 7 in the Supplement). The results of sensitivity analyses were similar to those of the main analysis.

### Individual-Level Multidomain Factors Associated With Glycemic Control Trajectories

The point estimates of the multidomain factors remained similar, with attenuation after sequential adjustment for the multidomain factors in models 1, 2, and 3 (eTable 8 in the Supplement). In the fully adjusted model 3, individuals in T1 were less likely to be older or to be Black or other race or ethnicity, whereas individuals in T3 or T4 were more likely to be Black compared with individuals in T2 (Table 2). Individuals in T1 were less likely to have Medicaid or Medicare during pregnancy, while those in T4 were more likely to be in the second or third quartile of neighborhood poverty level and multiparous. Compared with individuals in T2, those in T1 were less likely to have overweight or obesity before pregnancy and to smoke during pregnancy, whereas those in T4 were less likely to have underweight and more likely to have overweight or obesity before pregnancy and to smoke during pregnancy. Individuals in T3 and T4, compared with those in T2, were more likely to consume alcohol during pregnancy (Table 2).

**Table 2.** Associations of Individual-Level Multidomain Factors With Each Glycemic Control Trajectory

Factor	Adjusted relative risk (95% CI)^a^		
	T1	T3	T4
Age at delivery, y			
18-24	1 [Reference]	1 [Reference]	1 [Reference]
25-29	0.92 (0.87-0.98)^b^	0.96 (0.84-1.10)	1.03 (0.88-1.21)
30-34	0.87 (0.82-0.92)^b^	0.97 (0.85-1.11)	1.00 (0.86-1.17)
35-55	0.82 (0.78-0.87)^b^	0.97 (0.85-1.10)	1.02 (0.87-1.20)
Race and ethnicity			
Asian/Pacific Islander	0.97 (0.94-1.01)	1.00 (0.94-1.07)	1.01 (0.93-1.10)
Black	0.84 (0.76-0.92)^b^	1.17 (1.04-1.31)^b^	1.24 (1.08-1.41)^b^
Hispanic	0.98 (0.94-1.02)	0.98 (0.91-1.05)	1.00 (0.91-1.09)
White	1 [Reference]	1 [Reference]	1 [Reference]
Other^c^	0.88 (0.81-0.96)^b^	0.99 (0.86-1.13)	1.10 (0.93-1.29)
Neighborhood poverty level, % below threshold^c^			
1 (0-4, lowest)	1 [Reference]	1 [Reference]	1 [Reference]
2 (5-9)	1.00 (0.97-1.03)	1.00 (0.93-1.06)	1.11 (1.02-1.20)^b^
3 (10-19)	1.00 (0.96-1.03)	0.99 (0.92-1.05)	1.10 (1.02-1.20)^b^
4 (≥20, highest)	0.99 (0.95-1.04)	0.94 (0.87-1.02)	1.00 (0.90-1.11)
Medicare/Medicaid during pregnancy			
No	1 [Reference]	1 [Reference]	1 [Reference]
Yes	0.91 (0.86-0.96)^b^	0.93 (0.85-1.01)	1.07 (0.97-1.17)
Multiparity			
No	1 [Reference]	1 [Reference]	1 [Reference]
Yes	1.02 (0.99-1.05)	1.01 (0.96-1.07)	1.12 (1.05-1.20)^b^
Pre-pregnancy BMI^d^			
<18.5	1.06 (0.98-1.14)	0.77 (0.55-1.08)	0.48 (0.25-0.92)^b^
18.5-24.9	1 [Reference]	1 [Reference]	1 [Reference]
25.0-29.9	0.86 (0.83-0.89)^b^	1.03 (0.95-1.11)	1.22 (1.10-1.37)^b^
≥30.0	0.69 (0.67-0.72)^b^	1.00 (0.93-1.08)	1.41 (1.27-1.57)^b^
Smoking during pregnancy			
No	1 [Reference]	1 [Reference]	1 [Reference]
Yes	0.84 (0.75-0.93)^b^	0.86 (0.73-1.01)	1.27 (1.11-1.46)^b^
Alcohol during pregnancy			
No	1 [Reference]	1 [Reference]	1 [Reference]
Yes	0.97 (0.91-1.02)	1.14 (1.04-1.25)^b^	1.14 (1.02-1.28)^b^
Higher OGTT glucose levels^e^			
No	1 [Reference]	1 [Reference]	1 [Reference]
Yes	0.69 (0.65-0.73)^b^	1.02 (0.95-1.09)	1.32 (1.22-1.42)^b^
Early GDM diagnosis^f^			
No	1 [Reference]	1 [Reference]	1 [Reference]
Yes	1.04 (1.00-1.07)	1.85 (1.75-1.95)^b^	1.72 (1.61-1.83)^b^
RPSC program engagement^g^			
No	1 [Reference]	1 [Reference]	1 [Reference]
Yes	1.02 (0.97-1.06)	0.82 (0.76-0.90)^b^	0.68 (0.62-0.76)^b^
Frequency of SMBG measurements tertile (times/d)			
1 (≤2)	1 [Reference]	1 [Reference]	1 [Reference]
2 (3)	1.00 (0.96-1.03)	0.86 (0.81-0.92)^b^	0.69 (0.64-0.74)^b^
3 (≥4)	1.02 (0.99-1.06)	0.68 (0.63-0.72)^b^	0.46 (0.42-0.50)^b^

Abbreviations: BMI, body mass index (calculated as weight in kilograms divided by height in meters squared); GDM, gestational diabetes; OGTT, oral glucose tolerance test; RPSC, Regional Perinatal Service Center; SMBG, self-monitoring of blood glucose; T1, stably optimal trajectory; T3, slowly improving to near-optimal trajectory; and T4, slowly improving to suboptimal trajectory.

^a^ Data are calculated using Poisson regression models with robust SEs, with the rapidly-improving-to-optimal glycemic control trajectory as the reference group for the given trajectory outcome. The model was adjusted for age at delivery, race and ethnicity, neighborhood poverty level, Medicare or Medicaid during pregnancy, nulliparity, pre-pregnancy body mass index, smoking and alcohol during pregnancy, higher OGTT glucose levels, early GDM diagnosis, RPSC program engagement, and frequency of SMBG measurements.

^b^ False discover rate–corrected *P* < .05.

^c^ Other race and ethnicity includes American Indian or Alaskan Native, multiracial, and missing race and ethnicity.

^d^ Defined as the percentage of households in neighborhood below the poverty level, divided into quartiles.

^e^ Higher OGTT glucose levels were defined as at least 2 of the fasting, 1-hour, 2-hour, and 3-hour glucose levels during the 100-g, 3-hour oral glucose tolerance test more than 1 SD above the respective population mean.

^f^ Early GDM diagnosis was defined as diagnosis of GDM before 24 weeks of gestation.

^g^ RPSC program engagement was defined as at least 1 call biweekly between enrollment into the RPSC GDM supplemental care program and delivery.

As for indicators of gestational diabetes severity and management adherence, individuals in T1 were less likely while individuals in T4 were more likely to have higher glucose levels during the diagnostic OGTT, compared with T2. Individuals in T3 or T4 were more likely to have early gestational diabetes diagnosis before 24 weeks of gestation and less likely to engage in at least 1 call biweekly with RPSC nurses and conduct self-monitoring of blood glucose measurements at least 3 times a day.

## Discussion

In a population-based, racially and ethnically diverse cohort study of 26 774 individuals with gestational diabetes, we identified 4 glycemic control trajectories between diagnosis and delivery: T1, stably optimal; T2, rapidly improving to optimal; T3, slowly improving to near-optimal; and T4, slowly improving to suboptimal, which were associated with perinatal complications with a gradient. Specifically, the risks of cesarean delivery, shoulder dystocia, LGA, and NICU admission were lower in T1 and higher in T4 compared with T2, with SGA risk following an opposite pattern. Taken together, our findings highlight the importance of achieving glycemic control early after gestational diabetes diagnosis and throughout pregnancy to decrease the risk of perinatal complications.

Trajectory analysis using latent class modeling is an innovative semiparametric statistical method to identify distinct patterns over time using longitudinal data.^18^ Prior studies on glycemic control trajectories among nonpregnant individuals used data on glycated hemoglobin (HbA_1c_) during transition to adolescence,^23^ between adolescence and adulthood^24^ or during adulthood.^25^ However, during pregnancy, the ADA recommends that self-monitoring of blood glucose, not HbA_1c_, should be used as the primary measure of glycemic control due to the physiological increase in red blood cell turnover leading to lower HbA_1c_ levels.^6^ Further, HbA_1c_ represents an integrated measure of glucose, not fully capturing postprandial hyperglycemia,^26,27^ which is an important pathophysiologic aspect of gestational diabetes.^28^ HbA_1c_ is a measure of glycemic control during the previous 8 to 12 weeks^29^ and thus is limited in its ability to examine hyperglycemia in a timely manner for gestational diabetes, which is usually diagnosed at 24 to 28 weeks of gestation, leaving only 12 to 16 weeks from diagnosis to delivery.

We identified a gradient of increasing risk of perinatal complications overall across T1 to T4 glycemic control trajectories. In previous interventions aiming to improve gestational diabetes outcomes through lifestyle modification or medications,^4,5,30,31,32,33,34^ some but not all interventions reported decreased risk of perinatal complications.^30,31,33^ Importantly, few studies reported attainment of glycemic control and, to our knowledge, none examined the direct association of glycemic control via self-monitoring of blood glucose with the risk of perinatal complications. In this regard, our findings contribute to the literature by illustrating the direct associations of glycemic control trajectories with the risk of perinatal complications. Individuals in T1 and T2 were more likely to conduct self-monitoring of blood glucose measurements; thus, the accuracy of classification in these 2 trajectories may be higher than in T3 and T4. However, the possible misclassification errors in T3 and T4 may have attenuated the true associations between the glycemic control trajectories and perinatal complications, resulting in underestimated effect sizes. Of note, we observed a decreasing trend of SGA risk across T1 to T4; therefore, more efforts are needed to improve glycemic control while mitigating the risk of SGA.

Several individual-level multidomain factors were associated with distinct glycemic control trajectories. Compared with individuals in T2, individuals in T3 and T4 were more likely to be Black, consume alcohol during pregnancy, and have gestational diabetes diagnosis before 24 weeks of gestation and less likely to adhere to gestational diabetes management (ie, engaging in ≥1 counseling call with RPSC nurses biweekly and conducting self-monitoring of blood glucose ≥3 times/d). Findings from this study provide a better understanding of the subgroups of individuals with gestational diabetes following distinct glycemic control trajectories and their individual-level characteristics. This may allow for risk stratification of gestational diabetes–complicated pregnancies and inform future multicomponent interventions to achieve glycemic control early after gestational diabetes diagnosis and throughout pregnancy to decrease the risk of perinatal complications. Specifically, our findings can support efforts targeting modifiable risk factors, such as management of overweight and obesity among individuals of child-bearing age and engagement in self-monitoring of blood glucose among individuals with gestational diabetes.

### Strengths and Limitations

This study has several strengths. To our knowledge, this is among the first population-based studies to profile glycemic control trajectories based on serial self-monitoring of blood glucose measurements from gestational diabetes diagnosis through delivery. Glycemic control trajectories uniquely reflect dynamic changes in glycemic control over time, which present valuable information on both the magnitude and duration of glycemic control exposure compared with a dichotomous glycemic control status (optimal vs suboptimal). In addition, our study population is racially and ethnically diverse, improving generalizability of our findings. Furthermore, universal screening for gestational diabetes and universal referral to the RPSC supplemental care program for individuals with gestational diabetes minimize misclassification bias and variations in access to health care and clinical practice, which have been major methodological limitations in the literature.

Our study has some limitations. Although we controlled for an array of covariates, residual confounding cannot be ruled out. For instance, we did not have data on individuals’ dietary intake or physical activity during pregnancy. However, diet and physical activity counseling are the major components of the RPSC supplemental gestational diabetes care program,^35^ and adjustment for them may result in over adjustment, given that we have included indicators of gestational diabetes management adherence in the final model. We excluded individuals who had diabetes prior to pregnancy but not individuals with prediabetes prior to the index pregnancy because prediabetes and earlier blood glucose control may be precursors to gestational diabetes per ADA guidelines,^6^ and individuals of reproductive age are not universally screened for prediabetes. We used self-monitoring of blood glucose measurements routinely collected in clinic practice, which were subject to self-report bias and inherent intraindividual variability that would mostly introduce random errors rather than systematic bias. However, these data reflect what is currently used in clinical practice to manage glycemic control; thus, the results are directly applicable to the health care setting. Furthermore, these variabilities would result in more conservative or underestimated effect sizes of associations, and the use of serial self-monitoring of blood glucose measurements during pregnancy to profile glycemic control trajectories would reduce random measurement errors.

## Conclusions

This cohort study found that slowly improving glycemic control trajectories following gestational diabetes diagnosis were associated with increased risk of perinatal complications. Several sociodemographic, clinical, lifestyle, and gestational diabetes–related multidomain factors were associated with slowly improving glycemic control trajectories. Future interventions should help individuals achieve glycemic control early after gestational diabetes diagnosis and throughout pregnancy to decrease the risk of perinatal complications. Early identification of individuals with gestational diabetes with the characteristics associated with distinct glycemic control trajectories may inform a risk stratification–based model of care for gestational diabetes, serving as a critical step toward precision medicine. Future studies exploring the long-term outcomes of distinct glycemic control trajectories as well as barriers and facilitators to achieving optimal glycemic control trajectories are warranted.

## Notes

Supplement. **eAppendix.** Trajectory Modeling **eFigure.** Actual and Estimated Glycemic Control Trajectories Between Gestational Diabetes Diagnosis and Delivery **eTable 1.** Trajectory Modeling Fit Criteria **eTable 2.** Comparison of Characteristics of Individuals with Gestational Diabetes in the Analytical Sample versus Those Excluded Due to Missing Data on Self-monitoring of Blood Glucose **eTable 3.** Distributions of Self-monitored Blood Glucose Measurements Among Individuals With Gestational Diabetes Who Received Prenatal Care in 2007-2017 at Kaiser Permanente Northern California **eTable 4.** Association of Glycemic Control Trajectories With Perinatal Complications Using the Rapidly Improving to Optimal GC Trajectory as Reference Group **eTable 5.** Association of Glycemic Control Trajectories With Perinatal Complications Using the Stably Optimal GC Trajectory as Reference Group **eTable 6.** Association of Glycemic Control Trajectories With Perinatal Complications Adjusting for Gestational Weight Gain **eTable 7.** Association of Glycemic Control Trajectories With Perinatal Complications Excluding Individuals With Early Gestational Diabetes Diagnosis Before 24 Weeks Gestation **eTable 8.** Associations of Patient-Level Multidomain Factors With Each Glycemic Control Trajectory Compared With the Rapidly Improving to Optimal Trajectory
