# Supplementary material for: Glycemic Control Trajectories and Risk of Perinatal Complications Among Individuals With Gestational Diabetes
Source: JAMA Netw Open. 2022 Sep 29;5(9):e2233955. doi: 10.1001/jamanetworkopen.2022.33955 (PMC9523493; doi:10.1001/jamanetworkopen.2022.33955)
Supplement: Supplement. — eAppendix. Trajectory Modeling eFigure. Actual and Estimated Glycemic Control Trajectories Between Gestational Diabetes Diagnosis and Delivery eTable 1. Trajectory Modeling Fit Criteria eTable 2. Comparison of Characteristics of Individuals with Gestational Diabetes in the Analytical Sample versus Those Excluded Due to Missing Data on Self-monitoring of Blood Glucose eTable 3. Distributions of Self-monitored Blood Glucose Measurements Among Individuals With Gestational Diabetes Who Received Prenatal Care in 2007-2017 at Kaiser Permanente Northern California eTable 4. Association of Glycemic Control Trajectories With Perinatal Complications Using the Rapidly Improving to Optimal GC Trajectory as Reference Group eTable 5. Association of Glycemic Control Trajectories With Perinatal Complications Using the Stably Optimal GC Trajectory as Reference Group eTable 6. Association of Glycemic Control Trajectories With Perinatal Complications Adjusting for Gestational Weight Gain eTable 7. Association of Glycemic Control Trajectories With Perinatal Complications Excluding Individuals With Early Gestational Diabetes Diagnosis Before 24 Weeks Gestation eTable 8. Associations of Patient-Level Multidomain Factors With Each Glycemic Control Trajectory Compared With the Rapidly Improving to Optimal Trajectory [file jamanetwopen-e2233955-s001.pdf]

Supplemental Online Content

Chehab RF, Ferrara A, Greenberg MB, Ngo AL, Feng J, Zhu Y. Glycemic control trajectories and risk of perinatal complications among individuals with gestational diabetes. *JAMA Netw Open*. 2022;5(9):e2233955. doi:10.1001/jamanetworkopen.2022.33955

eAppendix. Trajectory Modeling
eFigure. Actual and Estimated Glycemic Control Trajectories Between Gestational Diabetes Diagnosis and Delivery
eTable 1. Trajectory Modeling Fit Criteria
eTable 2. Comparison of Characteristics of Individuals with Gestational Diabetes in the Analytical Sample versus Those Excluded Due to Missing Data on Self-monitoring of Blood Glucose
eTable 3. Distributions of Self-monitored Blood Glucose Measurements Among Individuals With Gestational Diabetes Who Received Prenatal Care in 2007-2017 at Kaiser Permanente Northern California
eTable 4. Association of Glycemic Control Trajectories With Perinatal Complications Using the Rapidly Improving to Optimal GC Trajectory as Reference Group
eTable 5. Association of Glycemic Control Trajectories With Perinatal Complications Using the Stably Optimal GC Trajectory as Reference Group
eTable 6. Association of Glycemic Control Trajectories With Perinatal Complications Adjusting for Gestational Weight Gain
eTable 7. Association of Glycemic Control Trajectories With Perinatal Complications Excluding Individuals With Early Gestational Diabetes Diagnosis Before 24 Weeks Gestation
eTable 8. Associations of Patient-Level Multidomain Factors With Each Glycemic Control Trajectory Compared With the Rapidly Improving to Optimal Trajectory

This supplemental material has been provided by the authors to give readers additional information about their work.

© 2022 Chehab RF et al. *JAMA Network Open.*

eAppendix Trajectory Modeling

We derived glycemic control trajectories using latent class modeling,[1] which was fit using SAS Proc Traj (version 2020).[2,3] The models identified subgroups of individuals with GDM with similar underlying trajectories of serial self-monitoring of blood glucose measurements between GDM diagnosis and delivery. The time intervals were two gestational weeks, during which we examined whether the individual achieved optimal glycemic control. We assessed model fit of models with different numbers and forms of potential trajectories and identified that the model with four distinct glycemic control trajectories fits best based on Bayesian Information Criterion and clinical interpretability. In our final model, one group had a quadratic order term and three had linear order terms. Study individuals were assigned to the trajectory group for which they had the greatest posterior predictive probability (range 74%-86%; **Supplemental Table 1**).

References:

[1]Nagin D. *Group-Based Modeling of Development.* Harvard University Press; 2005.

[2]Jones BL. Traj: group-based modeling of longitudinal data. . Accessed 2021, https://www.andrew.cmu.edu/user/bjones/

[3]Jones BL, Nagin DS, Roeder K. A SAS procedure based on mixture models for estimating developmental trajectories. *Sociological methods & research*. 2001;29(3):374-393.

© 2022 Chehab RF et al. *JAMA Network Open.*

eFigure. Actual and Estimated Glycemic Control Trajectories Between Gestational Diabetes Diagnosis and Delivery

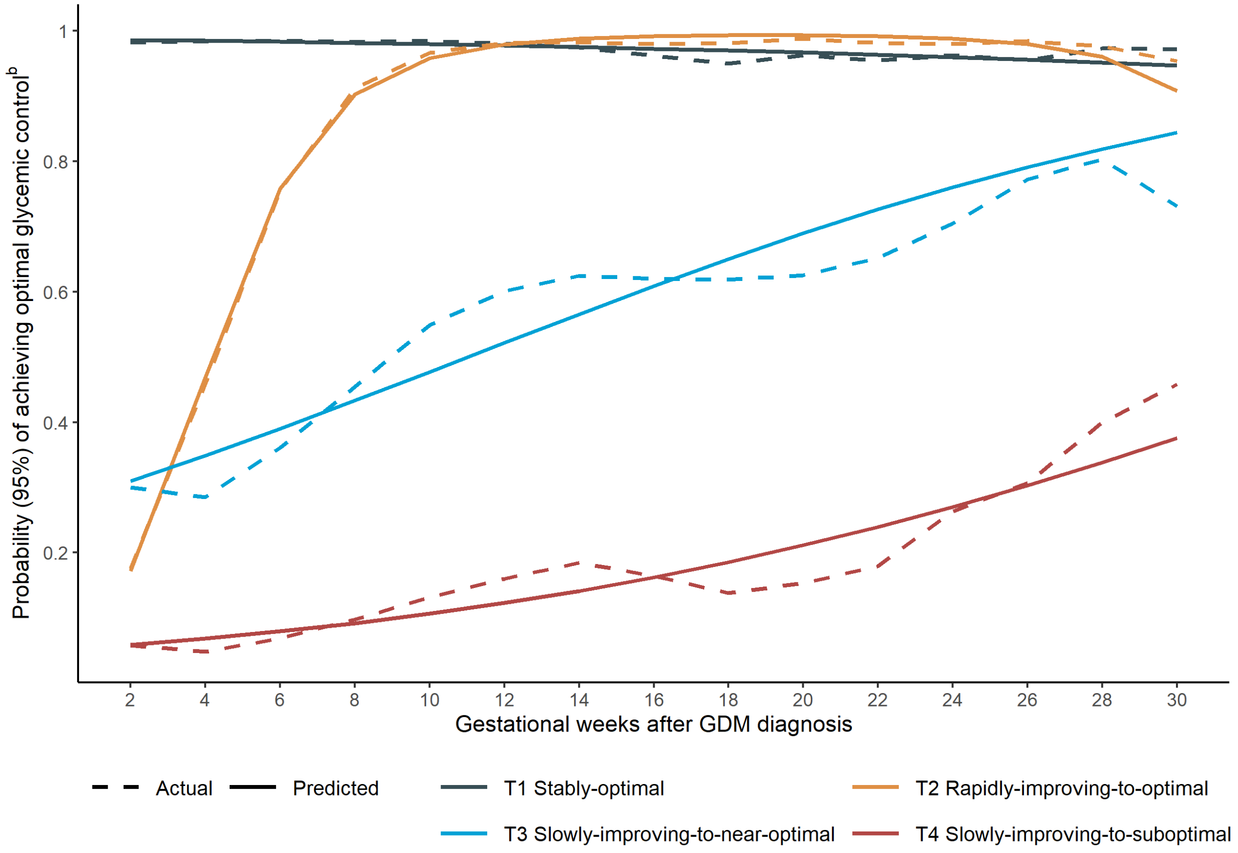

T1-T4, trajectories 1-4.

[a]Trajectories T1-T4 were derived using serial self-monitored blood glucose measurements between gestational diabetes diagnosis and delivery.

[b]Optimal glycemic control was defined as ≥80% of all self-monitored blood glucose measurements meeting the targets recommended by the American Diabetes Association guidelines and implemented at Kaiser Permanente Northern California.

© 2022 Chehab RF et al. *JAMA Network Open.*

eTable 1. Trajectory Modeling Fit Criteria

		Posterior predictive probability			
	**n (%)**	**Mean**	**SD**	**Minimum**	**Maximum**
**T1 Stably-optimal GC trajectory**	10528 (39.3)	0.86	0.12	0.37	0.98
**T2 Rapidly-improving-to-optimal GC trajectory**	9151 (34.2)	0.80	0.17	0.35	1.00
**T3 Slowly-improving-to-near-optimal GC trajectory**	4161 (15.5)	0.74	0.18	0.36	1.00
**T4 Slowly-improving-to-suboptimal GC trajectory**	2934 (11.0)	0.74	0.18	0.38	1.00

GC, glycemic control; SD, standard deviation.

© 2022 Chehab RF et al. *JAMA Network Open.*

eTable 2. Comparison of Characteristics of Individuals with Gestational Diabetes in the Analytical Sample versus Those Excluded Due to Missing Data on Self-monitoring of Blood Glucose

**Patient-level multidomain factors**	**Analytical sample (N = 26,774)**	**Individuals with GDM not enrolled in RPSC (N = 2,099)**	***P*-value**
	n (%)	n (%)	
**Age at delivery, years**			
18-24	1313 (4.9)	208 (9.9)	<0.0001
25-29	5319 (19.9)	458 (21.8)	
30-34	10002 (37.4)	703 (33.5)	
35-55	10140 (37.9)	730 (34.8)	
**Race/ethnicity**			
White	6049 (22.6)	438 (20.9)	<0.0001
Asian/Pacific Islander	11196 (41.8)	686 (32.7)	
Black	1083 (4.0)	186 (8.9)	
Hispanic	7500 (28.0)	711 (33.9)	
Other[a]	946 (3.5)	78 (3.7)	
**Multiparity**	15738 (58.8)	1359 (64.7)	<0.0001
**Pre-pregnancy BMI**[b]			
Underweight	352 (1.3)	25 (1.2)	<0.0001
Normal	6087 (22.7)	323 (15.4)	
Overweight	8810 (32.9)	656 (31.3)	
Obese	11525 (43.0)	1095 (52.2)	
**Smoking during pregnancy**	672 (2.5)	77 (3.7)	<0.0001
**Alcohol during pregnancy**	1732 (6.5)	127 (6.1)	<0.0001
**Higher OGTT glucose levels**[c]	3121 (11.7)	271 (12.9)	0.086

BMI, body mass index; OGTT, oral glucose tolerance test; RPSC, regional perinatal service center; SMBG, self-monitoring of blood glucose.

P-value calculated using Chi-square test.

[a]Other race and ethnicity includes American Indian/Alaskan Native, multiracial, and missing race and ethnicity.

[b]Pre-pregnancy BMI: underweight (<18.5 kg/m^2), normal (18.5-24.9 kg/m^2), overweight (25.0-29.9 kg/m^2), obese (≥30.0 kg/m^2).

[c]Higher OGTT glucose levels: at least two of the fasting, 1-hour, 2-hour, and 3-hour glucose levels during the 100-g, 3-hour oral glucose tolerance test >1 standard deviation above the respective population mean.

© 2022 Chehab RF et al. *JAMA Network Open.*

eTable 3. Distributions of Self-monitored Blood Glucose Measurements Among Individuals With Gestational Diabetes Who Received Prenatal Care in 2007-2017 at Kaiser Permanente Northern California

	**Total (N = 26,774)**
Oral glucose tolerance test, mg/dL, mean ± SD	
Fasting	91.1 ± 12.1
1-hr	199.5 ± 24.1
2-hrs	176.0 ± 26.6
3-hrs	127.3 ± 33.2
Overall SMBG, *(n)*	*26,774*
Total duration, weeks	11.8 ± 6.6
Total count	239.6 ± 160.6
SMBG, fasting, *(n)*	*26,738*
Value, mg/dL, mean ± SD	87.4 ± 8.3
Count, mean ± SD	62.1 ± 40.7
SMBG, 1-hr post-breakfast, *(n)*	*26,674*
Value, mg/dL, mean ± SD	120.3 ± 11.8
Count, mean ± SD	59.1 ± 39.7
SMBG, 1-hr post-lunch, *(n)*	*26,653*
Value, mg/dL, mean ± SD	121.1 ± 10.3
Count, mean ± SD	57.8 ± 39.1
SMBG, 1-hr post-dinner, *(n)*	*26,632*
Value, mg/dL, mean ± SD	122.5 ± 11.4
Count, mean ± SD	57.9 ± 39.2
Hr, hour; SMBG, self-monitoring of blood glucose.	

© 2022 Chehab RF et al. *JAMA Network Open.*

eTable 4. Association of Glycemic Control Trajectories With Perinatal Complications Using the Rapidly Improving to Optimal GC Trajectory as Reference Group

Perinatal complications	T1 Stably-optimal GC trajectory	T2 Rapidly-improving-to-optimal GC trajectory	T3 Slowly-improving-to-near-optimal GC trajectory	T4 Slowly-improving-to-suboptimal GC trajectory	*P*-for-trend
	Adjusted relative risk (95% confidence interval)				
**Cesarean delivery**					
Model 1	0.92 (0.88, 0.96)[a]	1 (Reference)	1.02 (0.97, 1.07)	1.21 (1.15, 1.27)[a]	<0.0001
Model 2	0.93 (0.89, 0.96)[a]	1 (Reference)	1.01 (0.96, 1.06)	1.19 (1.14, 1.25)[a]	<0.0001
Model 3	0.93 (0.89, 0.96)[a]	1 (Reference)	1.01 (0.96, 1.06)	1.18 (1.12, 1.24)[a]	<0.0001
**Preterm birth**[b]					
Model 1	0.96 (0.88, 1.05)	1 (Reference)	0.97 (0.86, 1.09)	1.40 (1.25, 1.56)[a]	<0.0001
Model 2	0.98 (0.89, 1.07)	1 (Reference)	0.92 (0.82, 1.03)	1.32 (1.18, 1.48)[a]	0.0005
Model 3	0.98 (0.90, 1.07)	1 (Reference)	0.86 (0.77, 0.97) [a]	1.19 (1.06, 1.33)[a]	0.1010
**Spontaneous preterm birth**					
Model 1	0.94 (0.83, 1.06)	1 (Reference)	1.00 (0.85, 1.17)	1.36 (1.15, 1.60)[a]	0.0003
Model 2	0.95 (0.84, 1.08)	1 (Reference)	0.96 (0.82, 1.13)	1.31 (1.11, 1.54)[a]	0.0042
Model 3	0.95 (0.84, 1.08)	1 (Reference)	0.90 (0.77, 1.06)	1.17 (0.99, 1.38)	0.1317
**Medically-indicated preterm birth**					
Model 1	0.98 (0.86, 1.13)	1 (Reference)	0.93 (0.78, 1.10)	1.45 (1.23, 1.72)[a]	0.0013
Model 2	1.00 (0.87, 1.14)	1 (Reference)	0.84 (0.71, 1.00)	1.32 (1.12, 1.56)[a]	0.0880
Model 3	1.00 (0.88, 1.15)	1 (Reference)	0.79 (0.66, 0.94)[a]	1.19 (1.01, 1.41)[a]	0.6492
**Shoulder dystocia**					
Model 1	0.74 (0.60, 0.90)[a]	1 (Reference)	1.18 (0.94, 1.48)	1.49 (1.19, 1.87)[a]	<0.0001
Model 2	0.75 (0.61, 0.92)[a]	1 (Reference)	1.11 (0.88, 1.39)	1.40 (1.12, 1.76)[a]	<0.0001
Model 3	0.75 (0.61, 0.92)[a]	1 (Reference)	1.11 (0.88, 1.40)	1.41 (1.12, 1.78)[a]	<0.0001
**LGA vs. AGA**[c]					
Model 1	0.72 (0.67, 0.77)[a]	1 (Reference)	1.17 (1.08, 1.26)[a]	1.56 (1.45, 1.68)[a]	<0.0001
Model 2	0.74 (0.68, 0.79)[a]	1 (Reference)	1.13 (1.04, 1.22)[a]	1.49 (1.38, 1.60)[a]	<0.0001

© 2022 Chehab RF et al. *JAMA Network Open.*

Model 3	0.74 (0.69, 0.80)[a]	1 (Reference)	1.11 (1.02, 1.20)[a]	1.42 (1.31, 1.53)[a]	<0.0001
**SGA vs. AGA[c]**					
Model 1	1.11 (1.02, 1.21)[a]	1 (Reference)	0.95 (0.85, 1.07)	0.63 (0.53, 0.75)[a]	<0.0001
Model 2	1.10 (1.02, 1.20)[a]	1 (Reference)	0.93 (0.83, 1.05)	0.62 (0.52, 0.74)[a]	<0.0001
Model 3	1.10 (1.02, 1.20)[a]	1 (Reference)	0.94 (0.83, 1.05)	0.63 (0.53, 0.75)[a]	<0.0001
**NICU admission**					
Model 1	0.89 (0.82, 0.97)[a]	1 (Reference)	1.11 (1.01, 1.22)[a]	1.49 (1.35, 1.64)[a]	<0.0001
Model 2	0.89 (0.82, 0.97)[a]	1 (Reference)	1.05 (0.95, 1.15)	1.41 (1.28, 1.56)[a]	<0.0001
Model 3	0.90 (0.83, 0.97)[a]	1 (Reference)	1.01 (0.92, 1.12)	1.33 (1.20, 1.47)[a]	<0.0001
**NICU stay ≥7 days**					
Model 1	1.02 (0.89, 1.18)	1 (Reference)	0.94 (0.78, 1.12)	1.36 (1.14, 1.62)[a]	0.0457
Model 2	1.03 (0.90, 1.18)	1 (Reference)	0.86 (0.72, 1.03)	1.25 (1.05, 1.50)[a]	0.4033
Model 3	1.03 (0.90, 1.19)	1 (Reference)	0.79 (0.66, 0.95)[a]	1.08 (0.90, 1.30)	0.4661

AGA, appropriate-for-gestational age; GC, glycemic control; LGA, large-for-gestational age; NICU, neonatal intensive care unit; SGA, small for gestational age; T1-T4, trajectories 1-4.

Data are presented as adjusted relative risk (95% confidence interval) calculated using Poisson regression models with robust standard errors.

P-for-trend was calculated using the Poisson trend test.

Model 1 adjusted for age at delivery, race and ethnicity, neighborhood poverty level, Medicare/Medicaid during pregnancy, nulliparity, pre-pregnancy BMI, and smoking and alcohol during pregnancy.

Model 2 adjusted for factors in Model 1 in addition to higher OGTT glucose levels and early GDM diagnosis.

Model 3 adjusted for factors in Model 2 in addition to RPSC program engagement and frequency of SMBG measurements.

[a]$P_{FDR} < 0.05$.

[b]Preterm birth includes both subtypes: spontaneous and medically indicated preterm birth.

[c]AGA, LGA, SGA: Birth weight categories derived using sex and gestational age-specific percentiles calculated using a 2017 U.S. reference population.

© 2022 Chehab RF et al. *JAMA Network Open.*

eTable 5. Association of Glycemic Control Trajectories With Perinatal Complications Using the Stably Optimal GC Trajectory as Reference Group

**Perinatal complications**	**T1 Stably-optimal GC trajectory**	**T2 Rapidly-improving-to-optimal GC trajectory**	**T3 Slowly-improving-to-near-optimal GC trajectory**	**T4 Slowly-improving-to-suboptimal GC trajectory**	***P*-for-trend**
	Adjusted relative risk (95% confidence interval)				
**Cesarean delivery**	1 (Reference)	1.08 (1.04, 1.12)[a]	1.09 (1.03, 1.14)[a]	1.28 (1.21, 1.34)[a]	<0.0001
**Preterm birth**	1 (Reference)	1.02 (0.93, 1.11)	0.88 (0.78, 0.99)[a]	1.21 (1.07, 1.36)[a]	0.1010
**Shoulder dystocia**	1 (Reference)	1.34 (1.09, 1.64)[a]	1.48 (1.17, 1.89)[a]	1.88 (1.47, 2.40)[a]	<0.0001
**LGA vs. AGA**[b]	1 (Reference)	1.36 (1.26, 1.46)[a]	1.50 (1.38, 1.63)[a]	1.92 (1.76, 2.09)[a]	<0.0001
**SGA vs. AGA**[b]	1 (Reference)	0.91 (0.84, 0.98)[a]	0.85 (0.76, 0.95)[a]	0.57 (0.48, 0.68)[a]	<0.0001
**NICU admission**	1 (Reference)	1.11 (1.03, 1.21)[a]	1.13 (1.02, 1.25)[a]	1.48 (1.33, 1.64)[a]	<0.0001
**NICU stay ≥7 days**	1 (Reference)	0.97 (0.84, 1.11)	0.76 (0.64, 0.92)[a]	1.05 (0.87, 1.26)	0.4661

AGA, appropriate-for-gestational age; GC, glycemic control; LGA, large-for-gestational age; NICU, neonatal intensive care unit; SGA, small-for-gestational age; T1-T4, trajectories 1-4.

Data are presented as adjusted relative risk (95% confidence interval) calculated using Poisson regression models with robust standard errors.

P-for-trend was calculated using the Poisson trend test.

Model adjusted for age at delivery, race and ethnicity, neighborhood poverty level, Medicare/Medicaid during pregnancy, multiparity, pre-pregnancy BMI, smoking and alcohol during pregnancy, higher OGTT glucose levels, early GDM diagnosis, RPSC program engagement and frequency of SMBG measurements.

[a]P_{FDR} <0.05.

[b]AGA, LGA, SGA: Birth weight categories derived using sex and gestational age-specific percentiles calculated using a 2017 U.S. reference population.

© 2022 Chehab RF et al. *JAMA Network Open.*

eTable 6. Association of Glycemic Control Trajectories With Perinatal Complications Adjusting for Gestational Weight Gain

**Perinatal complications**	**T1 Stably-optimal GC trajectory**	**T2 Rapidly-improving-to-optimal GC trajectory**	**T3 Slowly-improving-to-near-optimal GC trajectory**	**T4 Slowly-improving-to-suboptimal GC trajectory**	***P*-for-trend**
	Adjusted relative risk (95% confidence interval)				
**Cesarean delivery**	0.94 (0.90 ,0.98)[a]	1 (Reference)	0.99 (0.95 ,1.04)	1.14 (1.08 ,1.20)[a]	<0.0001
**Preterm birth**	0.96 (0.88 ,1.05)	1 (Reference)	0.88 (0.78 ,0.99)[a]	1.26 (1.12 ,1.41)[a]	0.0049
**Shoulder dystocia**	0.77 (0.63 ,0.95)[a]	1 (Reference)	1.08 (0.86 ,1.36)	1.31 (1.04 ,1.65)[a]	<0.0001
**LGA vs. AGA**[b]	0.78 (0.72 ,0.83)[a]	1 (Reference)	1.07 (0.99 ,1.15)	1.29 (1.20 ,1.39)[a]	<0.0001
**SGA vs. AGA**[b]	1.07 (0.98 ,1.16)	1 (Reference)	0.96 (0.86 ,1.08)	0.68 (0.57 ,0.81)[a]	<0.0001
**NICU admission**	0.90 (0.83 ,0.97)[a]	1 (Reference)	1.01 (0.92 ,1.12)	1.33 (1.20 ,1.47)[a]	<0.0001
**NICU stay ≥7 days**	1.01 (0.88 ,1.16)	1 (Reference)	0.81 (0.67 ,0.97)[a]	1.16 (0.96 ,1.39)	0.9125

AGA, appropriate-for-gestational age; GC, glycemic control; LGA, large-for-gestational age; NICU, neonatal intensive care unit; SGA, small-for-gestational age; T1-T4, trajectories 1-4.

Data are presented as adjusted relative risk (95% confidence interval) calculated using Poisson regression models with robust standard errors.

P-for-trend was calculated using the Poisson trend test.

Model adjusted for age at delivery, race and ethnicity, neighborhood poverty level, Medicare/Medicaid during pregnancy, multiparity, pre-pregnancy BMI, gestation weight gain, smoking and alcohol during pregnancy, higher OGTT glucose levels, early GDM diagnosis, RPSC program engagement and frequency of SMBG measurements.

[a]P_{FDR} <0.05.

[b]AGA, LGA, SGA: Birth weight categories derived using sex and gestational age-specific percentiles calculated using a 2017 U.S. reference population.

© 2022 Chehab RF et al. *JAMA Network Open.*

eTable 7. Association of Glycemic Control Trajectories With Perinatal Complications Excluding Individuals With Early Gestational Diabetes Diagnosis Before 24 Weeks Gestation

Perinatal complications	T1 Stably-optimal GC trajectory	T2 Rapidly-improving-to-optimal GC trajectory	T3 Slowly-improving-to-near-optimal GC trajectory	T4 Slowly-improving-to-suboptimal GC trajectory	*P*-for-trend
	Adjusted relative risk (95% confidence interval)				
**Cesarean delivery**	0.91 (0.87 ,0.95)[a]	1 (Reference)	0.98 (0.93 ,1.05)	1.20 (1.13 ,1.28)[a]	<0.0001
**Preterm birth**	1.01 (0.91 ,1.12)	1 (Reference)	0.81 (0.69 ,0.94)[a]	1.15 (0.99 ,1.33)	0.9542
**Shoulder dystocia**	0.75 (0.60 ,0.95)[a]	1 (Reference)	1.11 (0.82 ,1.49)	1.45 (1.09, 1.94)[a]	<0.0001
**LGA vs. AGA**[b]	0.73 (0.67 ,0.80)[a]	1 (Reference)	1.09 (0.99 ,1.20)	1.37 (1.25 ,1.50)[a]	<0.0001
**SGA vs. AGA**[b]	1.16 (1.05 ,1.27)[a]	1 (Reference)	0.94 (0.81 ,1.08)	0.70 (0.58 ,0.86)[a]	<0.0001
**NICU admission**	0.94 (0.85 ,1.03)	1 (Reference)	0.96 (0.84 ,1.09)	1.37 (1.21 ,1.56)[a]	<0.0001
**NICU stay ≥7 days**	1.12 (0.95 ,1.32)	1 (Reference)	0.72 (0.56 ,0.93)[a]	1.13 (0.89 ,1.44)	0.1582

AGA, appropriate-for-gestational age; GC, glycemic control; LGA, large-for-gestational age; NICU, neonatal intensive care unit; SGA, small-for-gestational age; T1-T4, trajectories 1-4.

Data are presented as adjusted relative risk (95% confidence interval) calculated using Poisson regression models with robust standard errors.

P-for-trend was calculated using the Poisson trend test.

Model adjusted for age at delivery, race and ethnicity, neighborhood poverty level, Medicare/Medicaid during pregnancy, multiparity, pre-pregnancy BMI, smoking and alcohol during pregnancy, higher OGTT glucose levels, early GDM diagnosis, RPSC program engagement and frequency of SMBG measurements.

[a]P_{FDR} <0.05.

[b]AGA, LGA, SGA: Birth weight categories derived using sex and gestational age-specific percentiles calculated using a 2017 U.S. reference population.

© 2022 Chehab RF et al. *JAMA Network Open.*

eTable 8. Associations of Patient-Level Multidomain Factors With Each Glycemic Control Trajectory Compared With the Rapidly Improving to Optimal Trajectory

**Patient-level multidomain factors**	**T1 Stably-optimal GC trajectory**			**T3 Slowly-improving-to-near-optimal GC trajectory**			**T4 Slowly-improving-to-suboptimal GC trajectory**		
	Model 1	Model 2	Model 3	Model 1	Model 2	Model 3	Model 1	Model 2	Model 3
	Adjusted relative risk (95% confidence interval)								
**Age at delivery, years**									
18-24	1 (Reference)	1 (Reference)	1 (Reference)	1 (Reference)	1 (Reference)	1 (Reference)	1 (Reference)	1 (Reference)	1 (Reference)
25-29	0.93 (0.87, 0.98)[a]	0.92 (0.87, 0.98)[a]	0.92 (0.87, 0.98)[a]	0.96 (0.83, 1.10)	0.93 (0.81, 1.06)	0.96 (0.84, 1.10)	0.98 (0.83, 1.16)	0.95 (0.81, 1.12)	1.03 (0.88, 1.21)
30-34	0.87 (0.82, 0.92)[a]	0.87 (0.82, 0.92)[a]	0.87 (0.82, 0.92)[a]	0.98 (0.86, 1.12)	0.92 (0.81, 1.05)	0.97 (0.85, 1.11)	0.94 (0.80, 1.10)	0.89 (0.76, 1.04)	1.00 (0.86, 1.17)
35-55	0.83 (0.78, 0.88)[a]	0.83 (0.78, 0.88)[a]	0.82 (0.78, 0.87)[a]	1.02 (0.89, 1.16)	0.91 (0.80, 1.04)	0.97 (0.85, 1.10)	0.98 (0.84, 1.16)	0.89 (0.76, 1.04)	1.02 (0.87, 1.20)
**Race/ Ethnicity**									
White	1 (Reference)	1 (Reference)	1 (Reference)	1 (Reference)	1 (Reference)	1 (Reference)	1 (Reference)	1 (Reference)	1 (Reference)
Asian/Pacific Islander	0.97 (0.94, 1.00)	0.97 (0.94, 1.01)	0.97 (0.94, 1.01)	1.06 (0.99, 1.13)	1.00 (0.93, 1.07)	1.00 (0.94, 1.07)	1.05 (0.96, 1.14)	1.01 (0.92, 1.10)	1.01 (0.93, 1.10)
Black	0.82 (0.74, 0.90)[a]	0.83 (0.76, 0.92)[a]	0.84 (0.76, 0.92)[a]	1.22 (1.08, 1.37)[a]	1.22 (1.09, 1.38)[a]	1.17 (1.04, 1.31)[a]	1.39 (1.22, 1.59)[a]	1.37 (1.20, 1.57)[a]	1.24 (1.08, 1.41)[a]
Hispanic	0.98 (0.94, 1.02)	0.98 (0.94, 1.02)	0.98 (0.94, 1.02)	1.02 (0.94, 1.10)	0.99 (0.92, 1.06)	0.98 (0.91, 1.05)	1.07 (0.97, 1.17)	1.04 (0.95, 1.14)	1.00 (0.91, 1.09)
Other[b]	0.88 (0.81, 0.95)[a]	0.88 (0.81, 0.95)[a]	0.88 (0.81, 0.96)[a]	1.05 (0.91, 1.21)	0.99 (0.86, 1.14)	0.99 (0.86, 1.13)	1.19 (1.00, 1.41)	1.14 (0.97, 1.35)	1.10 (0.93, 1.29)
**Neighborhood poverty level, % below threshold[c]**									
Quartile 1 (0-4, lowest)	1 (Reference)	1 (Reference)	1 (Reference)	1 (Reference)	1 (Reference)	1 (Reference)	1 (Reference)	1 (Reference)	1 (Reference)
Quartile 2 (5-9)	1.00 (0.97, 1.03)	1.00 (0.97, 1.03)	1.00 (0.97, 1.03)	1.00 (0.93, 1.06)	1.00 (0.93, 1.06)	1.00 (0.93, 1.06)	1.11 (1.02, 1.21)[a]	1.11 (1.02, 1.20)[a]	1.11 (1.02, 1.20)[a]
Quartile 3 (10-19)	1.00 (0.96, 1.03)	1.00 (0.96, 1.03)	1.00 (0.96, 1.03)	0.99 (0.93, 1.06)	0.99 (0.92, 1.05)	0.99 (0.92, 1.05)	1.11 (1.02, 1.21)[a]	1.10 (1.02, 1.20)[a]	1.10 (1.02, 1.20)[a]
Quartile 4 (≥20, highest)	0.99 (0.94, 1.03)	0.99 (0.95, 1.03)	0.99 (0.95, 1.04)	0.96 (0.88, 1.05)	0.96 (0.88, 1.04)	0.94 (0.87, 1.02)	1.03 (0.92, 1.14)	1.02 (0.92, 1.13)	1.00 (0.90, 1.11)
**Medicare/Medicaid during pregnancy**									

© 2022 Chehab RF et al. *JAMA Network Open.*

No	1 (Reference)	1 (Reference)	1 (Reference)	1 (Reference)	1 (Reference)	1 (Reference)	1 (Reference)	1 (Reference)	1 (Reference)
Yes	0.91 (0.86, 0.96)[a]	0.91 (0.86, 0.96)[a]	0.91 (0.86, 0.96)[a]	0.96 (0.88, 1.05)	0.94 (0.87, 1.03)	0.93 (0.85, 1.01)	1.11 (1.01, 1.22)[a]	1.10 (1.00, 1.21)	1.07 (0.97, 1.17)
**Multiparity**									
No	1 (Reference)	1 (Reference)	1 (Reference)	1 (Reference)	1 (Reference)	1 (Reference)	1 (Reference)	1 (Reference)	1 (Reference)
Yes	1.02 (0.99, 1.05)	1.02 (0.99, 1.05)	1.02 (0.99, 1.05)	1.10 (1.04, 1.16)[a]	1.05 (0.99, 1.10)	1.01 (0.96, 1.07)	1.24 (1.16, 1.34)[a]	1.20 (1.12, 1.29)[a]	1.12 (1.05, 1.20)[a]
**Pre-pregnancy BMI**[d]									
Underweight	1.05 (0.97, 1.14)	1.06 (0.98, 1.14)	1.06 (0.98, 1.14)	0.76 (0.54, 1.07)	0.79 (0.56, 1.11)	0.77 (0.55, 1.08)	0.50 (0.26, 0.95)	0.50 (0.26, 0.94)[a]	0.48 (0.25, 0.92)[a]
Normal	1 (Reference)	1 (Reference)	1 (Reference)	1 (Reference)	1 (Reference)	1 (Reference)	1 (Reference)	1 (Reference)	1 (Reference)
Overweight	0.86 (0.83, 0.89)[a]	0.86 (0.83, 0.89)[a]	0.86 (0.83, 0.89)[a]	1.06 (0.98, 1.15)	1.03 (0.96, 1.11)	1.03 (0.95, 1.11)	1.29 (1.15, 1.44)[a]	1.25 (1.11, 1.39)[a]	1.22 (1.10, 1.37)[a]
Obese	0.69 (0.67, 0.71)[a]	0.69 (0.67, 0.72)[a]	0.69 (0.67, 0.72)[a]	1.15 (1.07, 1.23)[a]	1.01 (0.94, 1.09)	1.00 (0.93, 1.08)	1.64 (1.47, 1.82)[a]	1.46 (1.32, 1.63)[a]	1.41 (1.27, 1.57)[a]
**Smoking during pregnancy**									
No	1 (Reference)	1 (Reference)	1 (Reference)	1 (Reference)	1 (Reference)	1 (Reference)	1 (Reference)	1 (Reference)	1 (Reference)
Yes	0.83 (0.74, 0.93)[a]	0.83 (0.75, 0.93)[a]	0.84 (0.75, 0.93)[a]	0.89 (0.75, 1.06)	0.88 (0.74, 1.03)	0.86 (0.73, 1.01)	1.39 (1.20, 1.60)[a]	1.36 (1.18, 1.57)[a]	1.27 (1.11, 1.46)[a]
**Alcohol during pregnancy**									
No	1 (Reference)	1 (Reference)	1 (Reference)	1 (Reference)	1 (Reference)	1 (Reference)	1 (Reference)	1 (Reference)	1 (Reference)
Yes	0.97 (0.92, 1.03)	0.97 (0.91, 1.02)	0.97 (0.91, 1.02)	1.11 (1.01, 1.23)[a]	1.15 (1.04, 1.27)[a]	1.14 (1.04, 1.25)[a]	1.12 (1.00, 1.27)[a]	1.15 (1.02, 1.29)[a]	1.14 (1.02, 1.28)[a]
**Higher OGTT glucose levels**[e]									
No		1 (Reference)	1 (Reference)		1 (Reference)	1 (Reference)		1 (Reference)	1 (Reference)
Yes		0.69 (0.65, 0.73)[a]	0.69 (0.65, 0.73)[a]		1.02 (0.95, 1.10)	1.02 (0.95, 1.09)		1.33 (1.23, 1.44)[a]	1.32 (1.22, 1.42)[a]
**Early GDM diagnosis**[f]									
No		1 (Reference)	1 (Reference)		1 (Reference)	1 (Reference)		1 (Reference)	1 (Reference)
Yes		1.04 (1.01, 1.08)[a]	1.04 (1.00, 1.07)		1.77 (1.68, 1.87)[a]	1.85 (1.75, 1.95)[a]		1.59 (1.49, 1.70)[a]	1.72 (1.61, 1.83)[a]
**RPSC program engagement**[g]									

© 2022 Chehab RF et al. *JAMA Network Open.*

No			1 (Reference)			1 (Reference)			1 (Reference)
Yes			1.02 (0.97, 1.06)			0.82 (0.76, 0.90)[a]			0.68 (0.62, 0.76)[a]
**Frequency of SMBG measurements, times/day**									
Tertile 1 (≤2)			1 (Reference)			1 (Reference)			1 (Reference)
Tertile 2 (3)			1.00 (0.96, 1.03)			0.86 (0.81, 0.92)[a]			0.69 (0.64, 0.74)[a]
Tertile 3 (≥4)			1.02 (0.99, 1.06)			0.68 (0.63, 0.72)[a]			0.46 (0.42, 0.50)[a]

BMI, body mass index; GC, glycemic control; OGTT, oral glucose tolerance test; GDM, gestational diabetes; RPSC, Regional Perinatal Service Center; SMBG, self-monitoring of blood glucose; T1-T4, trajectories 1-4.
Data are presented as adjusted relative risk (95% confidence interval) calculated using Poisson regression models with robust standard errors with T2 rapidly-improving-to-optimal GC trajectory as the reference group for the given trajectory outcome.
Model 1 adjusted for age at delivery, race/ ethnicity, neighborhood poverty level, Medicare/Medicaid during pregnancy, nulliparity, pre-pregnancy BMI, and smoking and alcohol during pregnancy.
Model 2 adjusted for factors in Model 1 in addition to higher OGTT glucose levels and early GDM diagnosis.
Model 3 adjusted for factors in Model 2 in addition to RPSC program engagement and frequency of SMBG measurements.
[a] P_{FDR} <0.05.
[b] Other race and ethnicity includes American Indian/Alaskan Native, multiracial, and missing race and ethnicity.
[c] % of households in neighborhood below the poverty level, divided into quartiles.
[d] Pre-pregnancy BMI was categorized as follows: underweight (<18.5 kg/m^2), normal (18.5-24.9 kg/m^2), overweight (25.0-29.9 kg/m^2), obese (≥30.0 kg/m^2).
[e] Higher OGTT glucose levels: at least two of the fasting, 1-hour, 2-hour, and 3-hour glucose levels during the 100-g, 3-hour oral glucose tolerance test >1 standard deviation above the respective population mean.
[f] Early GDM diagnosis: diagnosis of GDM before 24 weeks of gestation.
[g] RPSC program engagement: the number of RPSC calls received ≥1 call biweekly between enrollment into the RPSC GDM supplemental care program and delivery.

© 2022 Chehab RF et al. *JAMA Network Open.*
